# Short-Wave Ultraviolet-Light-Based Disinfection of Surface Environment Using Light-Emitting Diodes: A New Approach to Prevent Health-Care-Associated Infections

**DOI:** 10.3390/microorganisms11020386

**Published:** 2023-02-02

**Authors:** Helena Duering, Thomas Westerhoff, Frank Kipp, Claudia Stein

**Affiliations:** 1 Institute for Infectious Diseases and Infection Control, Jena University Hospital, Am Klinikum 1, 07747 Jena, Germany; 2 Research Group “Smart UV Systems” at Fraunhofer Institute of Optronics, System Technologies and Image Exploitation, Am Vogelherd 90, 98693 Ilmenau, Germany

**Keywords:** UV-C LED irradiation, surface disinfection, hygiene, hospital acquired infections, reprocessing medical devices

## Abstract

Ultraviolet (UV)-C irradiation is a promising method for microbial eradication on surfaces. Major developments have taken place in UV-C light-emitting diodes (LEDs) technology. In this study, we examined the suitability of UV-C LED-based surface disinfection in hospitals. We tested the efficacy of UV-C LED surface treatment on different microorganisms dried on a carrier surface or in a liquid solution. The influences of soiling, shading, surface material, radiation wavelength, microbial load and species on the disinfection performance were investigated. UV-C LED caused a reduction of >5 log_10_ levels of *E. coli*, *S. aureus* and *C. albicans*, whereas 3 log_10_ reduction was observed for *G. stearothermophilus* spores. The components of the medium led to a reduced UV-C LED efficiency compared to buffered solutions. We observed that the microbial load and the roughness of the carrier surface had a major influence on the UV-C LED disinfection efficiencies, whereas shading had no impact on inactivation. This study showed that UV-C is suitable for surface disinfection, but only under certain conditions. We showed that the main factors influencing microbial inactivation through UV-C light (e.g., intrinsic and extrinsic factors) had a similar impact when using a UV-C LED radiation source compared to a conventional UV-C lamp. However, the potential of LEDs is contributed by their adjustable wavelength and customizable geometry for the decontamination of medical devices and surfaces, and thereby their ability to overcome shading effects.

## 1. Introduction

Increasing numbers of multidrug-resistant bacteria and nosocomial infections have posed major challenges to the global community [1]. To prevent infections and interrupt infection chains, new cleaning and disinfection methods are becoming the focus of research, especially for near-patient environments. Contactless ultraviolet (UV) irradiation can be used as a disinfectant treatment, and provides an alternative to conventional chemical disinfection. The most effective wavelengths of UV light for microbial inactivation are located between 200 nm and 280 nm—the short-wave ultraviolet (UV-C) [2]. Due to the high absorption of DNA, pyrimidine adducts and DNA–protein cross-links are generated upon irradiation, which inhibits the transcription and replication of DNA and thus leads to the inactivation of microorganisms [3,4]. Ultraviolet germicidal irradiation (UVGI) has long been known to be effective for air, water and food decontamination. On the one hand, microbiological characteristics influence the effectiveness of UV light; on the other hand, process parameters also have a significant impact on microbial inactivation. Intrinsic factors such as the type of microorganism, species and strain result in variation concerning repair efficiency, DNA condensation, pyrimidine content, cell size, cell wall thickness and pigmentation. The extrinsic factors include the growth phase of the microorganisms, growth conditions and recovery conditions after processing [4].

To date, most UV-C-based decontamination devices have used a conventional low-pressure mercury vapor lamp (LP) with an emission wavelength of 254 nm [5,6,7]. Recently, there have been major developments in the field of UV-C light-emitting diodes (LEDs). A great advantage of this new technology compared to traditional LP lamps is an adjustable wavelength optimized to the peak of maximum DNA absorption (265 nm). Additionally, LEDs are mercury-free, less temperature-dependent, do not require a warm-up period, are physically small and therefore can be arranged in a variety of designs and geometries. This is a particular advantage when irradiating complex surfaces, where shading is always a major factor in the success of microbial inactivation [8]. For these reasons, the technology offers many possible uses regarding surface disinfection, particularly in health care facilities.

Various surface compositions with partly complex geometries (especially in the case of medical devices) must be able to be disinfected quickly, cost-effectively and validated—even with high microbial loads—without damaging the surface materials. An example of this is the use of endoscopes, which are constantly becoming technically more sophisticated, more complex and therefore more expensive and challenging to clean. Since most endoscopes are used with high frequency in health care facilities, the reprocessing of the device before reuse plays an important role in the prevention of health-care-associated infections.

The aim of this study was to test the disinfection performance of UV-C LEDs on surfaces under standardized conditions and considering different clinically relevant aspects, and to evaluate whether UV-C LED radiation is suitable as a disinfection method.

## 2. Materials and Methods

### 2.1. Strains, Growth Conditions and Culture Preparation

Two bacterial strains, *Escherichia coli* ATCC 35218 and *Staphylococcus aureus* ATCC 29213, a *Candida albicans SC* 5314 yeast strain and commercially available *Geobacillus stearothermophilus* ATCC 7953 spore suspension (Merck KGaA, Darmstadt, Germany) were used. Microbial loads were set to 1 × 10^9^ to 5 × 10^9^ colony forming units (CFU)/mL for bacteria, 1 × 10^8^ to 5 × 10^8^ CFU/mL for yeast and 1 × 10^8^ CFU/mL for *G. stearothermophilus* spores.

### 2.2. UV-C LED Device

For the irradiation tests, a collimated-beam irradiation unit was developed, capable of homogeneously irradiating a circular area with a diameter of 25 mm. A UV-C LED of type S6060-DR250-W272-P100 (Bolb Inc., Livermore, CA, USA) with a peak emission wavelength of 272 nm (FWHM = 10 nm) and an optical power of 100 mW at 250 mA was used. The device was equipped with the LED, located at one end of a tube made of sintered optical PTFE 90 mm in length (distance from sample to radiation source). A daylight-blind SiC UV-C sensor of type SG01-18 from sgLux that detects the irradiation intensity at the exit surface was placed at the other end of the tube where the radiation exit surface was located.

**Figure 1:**
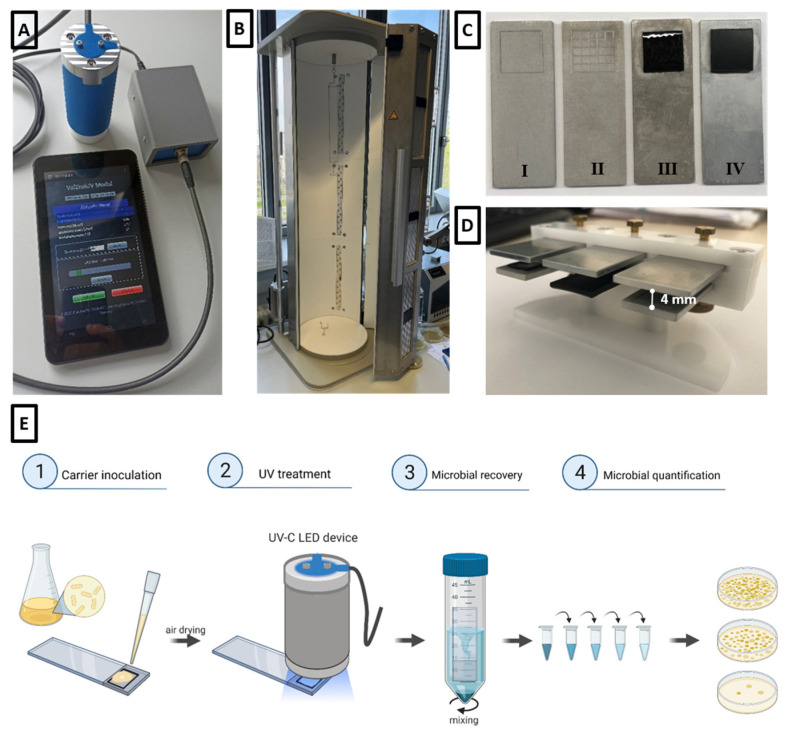
(**A**) Collimated-beam UV-C LED device with measurement and control units. (**B**) UV-C LED device for the irradiation of endoscopes with a cylindrical irradiation chamber. (**C**) Test carrier with test areas made of different materials. (I) Sandblasted aluminum smooth, (II) sandblasted aluminum textured, (III) elastomer and (IV) hard plastic. (**D**) Set-up for testing the influence of shading on UV-C efficiency. (**E**) Schematic diagram of the experimental setup and the different steps of the test procedure. Created with Biorender.com.

The UV-C sensor measured the actual irradiation intensity and integrated it into the dose over time with a resolution of 0.1 s. Once the target dose was reached, the LED automatically switched off. Figure 1A shows the collimated-beam UV-C LED device with the measurement and control unit.

Furthermore, we developed a UV-C light device suitable for the irradiation of endoscopes (see Figure 1B). It consisted of a cylindrical irradiation chamber with a length of 1000 mm and a diameter of 300 mm. The cylinder was vertical and consisted of two half shells. Twelve UV-C LED modules (six per half shell) with an individual length of 29 cm were integrated into the outer walls of the irradiation chamber at three different levels, radiating inwards. These UV-C LED modules were each equipped with 20 LEDs of type S6060-DR250-W272-P100 and thus with an individual radiant power of 2 watts at 272 nm. The total power of all 12 modules was thus 24 watts. Due to the design, the distance between the LED modules and the surface to be irradiated was between 10 cm (at the operating handle) and 14 cm (at the distal end). The inside of the irradiation chamber was fully lined with a coating of sintered optical PTFE, which is highly reflective (96% in the UV-C range) and also highly scattering. As a result, a very homogeneous radiation distribution inside the device and thus a homogeneous irradiation of the endoscope was achieved without shadowing, and with just a single reflection. To validate the irradiation process, three of the already mentioned SiC photosensors were installed in the device to measure the intensity in the irradiation room at the three heights of the irradiation modules. A display and a microcontroller unit were installed on the front of the device, which were used for operation. All measured values of the sensors as well as safety devices, which were intended to prevent accidental opening of the device and thus radiation leakage, were located on the front of the device.

### 2.3. Carrier Preparation

The test carriers were selected to represent the surfaces of clinical medical devices (see Figure 1C). A defined test area of 10 × 10 mm was engraved into the 40 × 15 mm aluminum test carrier (Figure 1(CI)). Additionally, the 10 × 10 mm test area of the aluminum carrier was textured by engraving a pattern to simulate material joints and connecting pieces (Figure 1(CII)). To test the effects of shading, the carriers were fixed in a holder and a plate was placed above them at a distance of 4 mm, as shown in Figure 1D.

### 2.4. Inoculation and UV Treatments

The experimental setup, as shown in Figure 1E, was developed and carried out in reference to the German DIN EN 14561:2006 for the evaluation of the bactericidal activity of chemical disinfectants and antiseptics for medical instruments. The inoculated test carrier was air-dried and directly used for UV irradiation at room temperature.

### 2.5. Microorganism Quantification and Reduction Calculation

After irradiation, the microorganisms were enumerated by spreading them onto tryptic soy agar (TSA) plates. Therefore, the test carrier was transferred to a 50 mL Falcon tube containing 10 mL phosphate buffer (0.25 M, pH 7.2) and 1 mL glass beads (0.5 mm diameter Carl Roth) and mixed for 1 min using a vortexer. After 5 min of incubation and remixing for 10 s, the sample was taken, diluted and plated onto TSA plates. The CFU were enumerated after the microorganisms were incubated on the plates for 24 h. With this experimental setup and an inoculation of 10^9^ CFU/mL, a 7 log_10_ reduction can be detected. A nonexposed control sample was included in each experiment.

### 2.6. Data Analysis

All experiments were conducted three times in duplicate. The inactivation performance for microorganisms was determined by counting the CFU, as described above, and presented as the mean log_10_ reduction and calculated using the following equation:

Log reduction = log × (*N*_0_/*N_t_*), (1)

where *N*_0_ is the concentration (CFU/mL) washed from the test carrier without UV treatment (nonexposed control sample) and *N_t_* is the concentration (CFU/mL) after UV irradiation. Statistical analysis was performed by using GraphPad Prism version 9.4, and a *p* value of <0.05 was considered significant.

## 3. Results

### 3.1. Comparison of the Disinfection Efficiency of UV-C LEDs for Liquid and Dried Surface Samples

**Figure 2:**
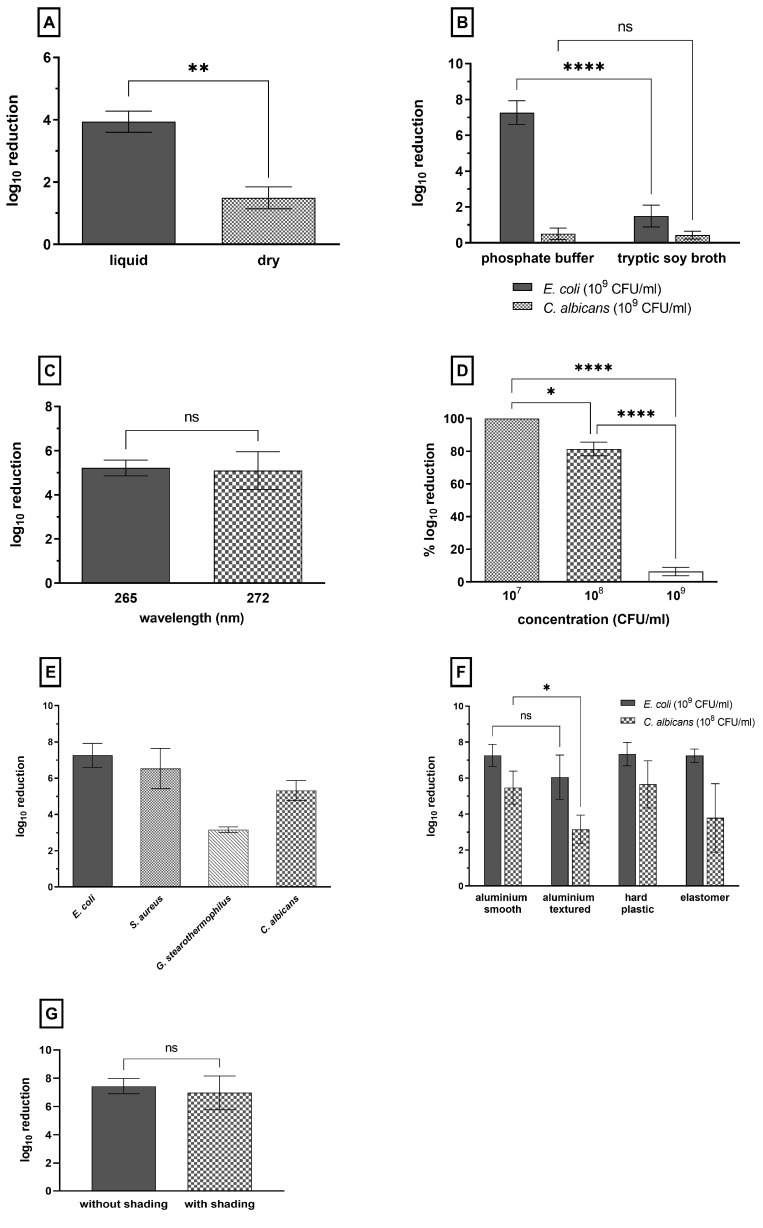
Log_10_ reduction of microorganisms under different conditions. (**A**) Comparison of the inactivation effects of UV-C treatment of suspension and surface-dried *E. coli* inoculated with 10^9^ CFU/mL after irradiation with 15,000 J/m^2^ at 272 nm in TSB. (**B**) Reduction of *E. coli* and *C. albicans* suspended in two different solutions as well as air-dried and exposed to 15,000 J/m^2^ at 272 nm. (**C**) Log_10_ inactivation of *E. coli* air-dried on a smooth aluminum surface and exposed to 200 J/m^2^ at different wavelengths. (**D**) Percent log_10_ reduction of different concentrations of *C. albicans* that were air-dried and treated with 15,000 J/m^2^ at 272 nm. (**E**) Inactivation performance for *E. coli*, *S. aureus* (1 × 10^9^ CFU/mL), *C. albicans* and *G. stearothermophilus* (1 × 10^8^ CFU/mL) resuspended in phosphate buffer and irradiated with 15,000 J/m^2^ using UV-C LED at 272 nm. (**F**) Comparison of the reduction of *E. coli* (10^9^ CFU/mL) and *C. albicans* (10^8^ CFU/mL) on different surface materials after treatment with UV-C at a dose of 15,000 J/m^2^. (**G**) Inactivation effects of UV-C treatment of *E. coli* (10^9^ CFU/mL) with and without shading after irradiation with 15,000 J/m^2^ at 272 nm. Data are presented as the mean ± standard deviation of three independent experiments performed in duplicate; *p* ≤ 0.0001 (****), *p* ≤ 0.01 (**), *p* ≤ 0.05 (*), *p* > 0.05 (ns).

To evaluate the disinfection performance of UV-C LEDs and their feasibility in routine clinical practice, we first compared the inactivation by irradiation of organisms in liquid, surrounding media and dried surface samples. We examined the log_10_ inactivation of the same bacterial load in a liquid droplet and dried on the surface of the test area. *E. coli* was inoculated into tryptic soy broth (TSB, a complex culture media) and exposed to 15,000 J/m^2^ of 272 nm UV-C light. In liquid surrounding media, *E. coli* was reduced by up to 4 log_10_, and with less than 5 log_10_ inactivation, no disinfection effect could be seen. On the dried surface, a 1.5 log_10_ reduction could be achieved, as shown in Figure 2A. However, the reduction in liquid surrounding media was >2 log_10_ higher than the reduction in the dried surface sample.

### 3.2. Influence of Surrounding Media and Nonmicrobial Contamination on the Disinfection Performance of UV-C Light

Figure 2B shows the reduction in disinfection performance due to absorption and shadowing effects from surrounding nonmicrobial organic and inorganic compounds. Whereas the *E. coli* suspended and air-dried in phosphate buffer was reduced by more than 7 log_10_ CFU/mL, the same strain suspended and air-dried in tryptic soy broth (TSB, a complex culture media) only showed a 1.5 log_10_ reduction. In comparison, the surrounding media showed no significant effect on the inactivation efficiency of *C. albicans* with a <1 log_10_ reduction in microbial load.

### 3.3. Comparison of Two Different Wavelengths in Regard to the Disinfection Efficiency of UV-C LEDs for E. coli on Surfaces

To investigate the effect of wavelength on disinfection performance, *E. coli* suspended in phosphate buffer and air-dried on a surface were exposed at 265 nm and 272 nm and treated with a dosage of 200 J/m^2^ (Figure 2C). No significant difference in the inactivation of *E. coli* was detected between both wavelengths.

### 3.4. Influence of the Microbial Load on UV-C LED Disinfection Performance (C. albicans)

The influence of the microbial load on the antimicrobial effect of UV-C LED light was tested using three different concentrations of *C. albicans* that were air-dried on the test area. *C. albicans* showed an inactivation of less than 1 log_10_ level after treatment with 15,000 J/m^2^ at a concentration of 10^9^ CFU/mL, regardless of the surrounding media. Figure 2D shows that inactivation of the microorganisms by 100% of the detectable log_10_ levels was achieved by reducing the concentration of the fungal load in two log_10_ steps from 10^9^ to 10^7^ CFU/mL. However, the detection limit of the test system was reduced along with the microbial load. With the usage of a 10^7^ CFU/mL microbial load, only a reduction of 5 log_10_ can be detected. A disinfection effect (>5 log_10_ reduction) could be achieved for *C. albicans* at a concentration of 10^8^ CFU/mL after treatment with 15,000 J/m^2^ and with an acceptable detection limit of 6 log_10_.

### 3.5. Effect of UV-C LED Irradiation on Different Microorganisms

To demonstrate disinfection, a dose of 15,000 J/m^2^ was required in the previously described experiments at a microbial load of 10^8^ CFU/mL and in the absence of interfering contaminants for a reduction of *C. albicans* by 5 log_10_. The purpose of this experiment was to test the reduction of other microorganisms under the same conditions, except for the microbial concentration. The reduction of *E. coli*, *S. aureus* as well as *C. albicans* at 15,000 J/m^2^ was >5 log_10_, whereas *G. stearothermophilus* exhibited a reduction of approximately 3 log_10_ (Figure 2E).

### 3.6. Effect of UV-C LED Irradiation on Different Surfaces

The correlation between the disinfection performance of UV-C radiation and the material and surface conditions of the irradiated surface was evaluated on the basis of four different test carriers, which mimicked the surfaces of medical devices. *E. coli* was tested with 10^9^ CFU/mL, and *C. albicans* was tested with 10^8^ CFU/mL.

*E. coli* could be reduced by >7 log_10_ on all surfaces, with the exception of the textured aluminum test area, where the reduction was slightly lower at 6 log_10_ (Figure 2F). *C. albicans* inactivation was >5 log_10_ for both smooth aluminum and hard plastic. In comparison, the reduction of the textured aluminum surface was 2 log_10_ lower than that on the smooth aluminum carrier. The mean value of inactivation of *C. albicans* on the elastomer test area was also only approximately 3 log_10_.

### 3.7. Effect of Shading on UV-C Efficiency

Shading is known to be a weak point of UV-C-based disinfection, so we compared the UV-C efficiency in case of a shaded and a non-shaded test carrier inoculated with *E. coli* (10^9^ CFU/mL) and exposed to 15,000 J/m^2^. Therefore, the UV-C device suitable for endoscope disinfection was used, and the carriers were adjusted in a test set-up, were a second plate (20 × 40 mm) was placed on top of the inoculated test carrier with a distance of 4 mm. We were able to demonstrate comparable inactivation effects for both test methods, with and without shading. In both cases, a reduction of >6 log_10_ was achieved, as shown in Figure 2G.

## 4. Discussion

### 4.1. Comparison of the Disinfection Efficiency of UV-C LEDs for Liquid and Dried Surface Samples

UV-C radiation has been used in water and liquid food treatment for decades, and so a great number of studies have already been carried out on its inactivation efficiency for microorganisms in liquids. A characteristic of shortwave, high-energy UV-C radiation is a very low penetration depth; therefore, only organisms that are directly exposed to the radiation are effectively inactivated [9,10,11]. The penetration of UV-C light is strongly influenced by the characteristics of the liquid, mainly the absorption coefficient [12]. In our test setup, the limiting factor was not the penetration depth because we only irradiated a droplet; the high absorption of the TSB medium was the main limitation. This high absorption explains the relatively low log_10_ reduction observed in this experiment with a high dosage of UV-C exposure compared to the inactivation levels of *E. coli* strains in solutions as well as surface-dried strains found in the literature. According to Hoyer (1998), a dosage of 280 J/m^2^ is required for 4 log_10_ inactivation of *E. coli* strains in drinking water disinfection [13]. Kim et al. (2002) found that *E. coli* resuspended in 0.1% peptone water and air-dried onto polished stainless steel could be reduced by more than 4 log_10_ levels after a dose of 150 J/m^2^ [14]. However, the conditions under which these experiments were conducted differed in terms of the species tested, bacterial load, surrounding media, surface material and UV-C source, making it difficult to compare the results. In any case, less data are available on surface disinfection, and in addition to the abovementioned factors, the structure and roughness of the tested surface play an important role in inactivation by irradiation, as shading events are known to be a weak point of UVGI [15]. However, the results of our UV-C LED source indicate a significantly reduced disinfection performance in terms of surface contamination compared to contamination in small volumes of liquid.

After air-drying, the bacteria sediment and form a multilayer on the surface, which is difficult to penetrate with UV-C. Air-drying leads to a concentration of these components due to the evaporation of the water content, which may protect the bacteria by absorption and shielding [4].

### 4.2. Influence of Surrounding Media and Nonmicrobial Contamination on the Disinfection Performance of UV-C Light

In comparison to *E. coli*, hardly any influence of interfering substances on the inactivation of *C. albicans* could be demonstrated. This suggests that the low disinfection performance at 15,000 J/m^2^ can be attributed to the properties of the organism itself. *C. albicans,* as a yeast species, differs fundamentally from bacteria in structure, size, pigmentation and capability for DNA repair. This suggests that the high resistance to UV-C is due to these properties, especially given the applied concentration of 1 × 10^9^ CFU/mL on the test area (10 × 10 mm).

Frequently touched surfaces in health care facilities vary highly in terms of initial microbial load, but also in the composition and concentration of inorganic and organic compounds [16]. Therefore, it is important to investigate the effect of nonmicrobial contaminants on the performance of UV-C LED irradiation, and to take this into consideration when implementing UV-C LED-based disinfection in clinical settings. In this study, the influence of interfering substances on the inactivation performance of UV-C was tested using TSB, a complex culture broth, and comparing its use to the use of phosphate buffer. Although TSB is not representative of soiling commonly found in the clinical environment (e.g., all types of body fluids or skin oil), the principal influence of interfering substances such as proteins, salts and glucose can nevertheless be investigated using this culture medium. In the case of *E. coli*, the negative impact of the broth’s components was demonstrated, and can be related to absorption, reflection and shadowing events by the components. These results are consistent with those found in the literature [3,17]. In 2021, Barancheshme et al. assessed the influence of saliva on the inactivation of *B. subtilis* spores by UV-C irradiation [18]. Their research group found that the inactivation of spores dried in artificial and human saliva after irradiation at 254 nm was reduced compared to spores suspended in phosphate saline. From these results, it can be stated that the eradication effect of UV-C strongly depends on the chemical components in the microbial surrounding. We were able to show that UV-C LEDs and LPs have similar dependencies and limitations for microbial inactivation depending on optical properties, mainly the UV absorbance and the turbidity of the fluid. Some components of chemical disinfectants (e.g., alcohol) are volatile and evaporate, and other compounds (e.g., glucoprotamine and surfactants) remain as chemical residues on the surfaces that influence the microbial environment. Further research is needed to estimate whether previous chemical cleaning and disinfection steps provide sufficient conditions for UV-C irradiation.

### 4.3. Comparison of Two Different Wavelengths in Regard to the Disinfection Efficiency of UV-C LEDs for E. coli on Surfaces

Although 265 nm is closer to the absorption maximum of DNA, the results of this study show no significant difference in the inactivation of *E. coli* for both wavelengths. Similar results were reported by Kim et al., who found no significant changes in the reduction of *E. coli* after treatment with 266 nm and 275 nm UV-C LEDs using a different experimental setup [19]. UV-C LED technology has developed rapidly in recent years, as it has many advantages over conventional UV-C mercury lamps. For example, LEDs do not require a warm-up time to reach maximum output, and the emitted wavelength can be precisely adjusted in the manufacturing process. However, LEDs with shorter wavelengths currently have a lower optical power output, are less electrically efficient (wall plug efficiency) and are more expensive [8]. For LED efficiency, huge progress can be expected (percentage of energy converted into UV-C light) in the coming years. Since no significant difference in the inactivation performance between the two wavelengths could be determined, the use of the currently cheaper and more powerful LEDs with 272 nm is reasonable.

### 4.4. Influence of the Microbial Load on UV-C LED Disinfection Performance (C. albicans)

The results of this study highlight the impact of microbial load on the disinfection performance of UV-C LED light. It is therefore recommended to combine UV-C LED disinfection with previous cleaning steps to reduce both nonmicrobial contamination and microbial load. As high microbial concentrations, large-sized cells and soiling are obviously weak points of a UV-C-based disinfection method [20,21,22], it should be considered that, depending on the intended application of the UV-C LED device, microbes could be concentrated in scratches or fissures by wiping. The same applies to junctions between different materials. The multilayered accumulation of organisms can then result in a reduced inactivation due to shielding and absorption events, especially in the case of physically larger microorganisms such as yeasts [23]. Williams et al. determined a microbial load of 3.3 × 10^6^ to 1.2 × 10^9^ CFU/mL in pus from endodontic dental abscesses, to name just one example of possible contamination levels [24]. In regard to the disinfection of medical equipment, very high microbial loads must be assumed for the validation of cleaning methods to ensure the successful reprocessing of medical devices and thus patient safety.

### 4.5. Effect of UV-C LED Irradiation on Different Microorganisms

In this study, the different UV-C LED susceptibilities of the tested microorganisms were demonstrated. The effectiveness of UV-C irradiation while using LEDs could thus be demonstrated, but it should be emphasized that this effect could only be achieved under certain conditions, such as a minimum of interfering organic and inorganic compounds. The Gram-negative bacterium *E. coli* showed the highest UV sensitivity, followed by the Gram-positive *S. aureus* and *C. albicans*. Cadnum et al. (2018) demonstrated that the UV sensitivity of microorganisms depends on the thickness and chemical structure of their cell walls, their ability to repair DNA damage and the structures of their DNA [25]. In their studies, Sommers et al. (2010) found a reduction of 3.6 log_10_ levels for an *S. aureus* strain dried on a bead-blasted stainless steel surface after irradiation with 1000 J/m^2^, and Kim et al. (2002) reduced *E. coli* by 5.3 log_10_ levels, also dried on stainless steel, using 600 J/m^2^ [14,26]. Although both investigations were carried out at a wavelength of 254 nm, with lower bacterial loads compared to this work, and had methodological differences by which the surviving bacteria were recovered from the surface, the results are similar to those observed in this study. The spores, on the other hand, had the highest resistance to UV-C. This result is partly consistent with that of Kim et al. (2017): their test results concluded in the same order of susceptibility, but their tests did not include spores [19].

### 4.6. Effect of UV-C LED Irradiation on Different Surfaces 

The results suggest that the inactivation performance of UV-C LED light depends on the character of the surface. Seams and joints on the surface reduce the disinfecting effect. The inactivation effect was attenuated in the case of the highly structured surfaces, and the reduction was decreased by 0.9 log_10_ and 2.3 log_10_ levels for *E. coli* and *C. albicans*. Limited eradication by UV-C must be expected for the decontamination of complex medical devices such as control panels with buttons.

### 4.7. Effect of Shading on UV-C Efficiency

From the results of the preliminary investigations, we developed a device suitable for the irradiation of endoscopes. The design of this device is intended to prevent possible shadowing by parts of the medical equipment, which is a weak point of UVGI since shading events result in a loss of inactivation efficiency [15]. We were able to show that shading directly above the test field, as is the case with complex medical products, had no adverse effect on the inactivation of the microorganisms. This effect results from the construction of the UV-C device and the arrangement of the LEDs. The result highlights the superiority of UV-C LEDs compared to conventional UV-C lamps, since shading is a bigger problem in that case.

## 5. Conclusions

The use of UV-C irradiation for the disinfection of surfaces in a patient-facing environment has been the subject of many research groups in recent years [5,6,25,27]. The results are promising, but difficult to compare. One of the major reasons for this is that, to date, there is no standardized detection method for validating the disinfection performance of UV-C irradiation on surfaces [28]. Through this study, we were able to demonstrate that the main factors influencing microbial inactivation through UV-C light, including intrinsic factors (microorganism, species) and extrinsic factors (growing conditions, bacterial load, media), had a similar impact when using a UV-C LED radiation source compared to a conventional LP lamp. 

In principle, UV-C LED-based surface disinfection can reduce germs, but only under the following conditions. The surface must be prepared by a manual cleaning step to eliminate interfering organic and/or inorganic compounds as well as massive microbial load. For use in the clinical environment, the areas in which these conditions can be met should therefore be carefully assessed before implementation so that UV-C LED disinfection can be used beneficially as a supplement to manual cleaning. However, the potential of the LEDs is offered by their adjustable wavelength and the optimal customizable geometry for the decontamination of medical devices and surfaces. Due to the arrangement of the LEDs, the radiation can penetrate into previously shaded areas and thus become effective in these as well.

## Data Availability

Not applicable.
